# Factors associated with mortality in HIV-infected and uninfected patients with pulmonary tuberculosis

**DOI:** 10.1186/1471-2458-9-409

**Published:** 2009-11-12

**Authors:** Ferdinand M Mugusi, Saurabh Mehta, Eduardo Villamor, Willy Urassa, Elmar Saathoff, Ronald J Bosch, Wafaie W Fawzi

**Affiliations:** 1 Department of Internal Medicine, Muhimbili University of Health and Allied Sciences, Dar es Salaam, Tanzania; 2 Department of Nutrition, Harvard School of Public Health, Boston, USA; 3 Department of Microbiology, Muhimbili University of Health and Allied Sciences, Dar es Salaam, Tanzania; 4 University of Munich, Munich, Germany; 5 Department of Biostatistics (RJB) and Global Health and Population (WWF), Harvard School of Public Health, Boston, USA

## Abstract

**Background:**

HIV has fuelled the TB epidemic in sub-Saharan Africa. Mortality in patients co-infected with TB and HIV is high. Managing factors influencing mortality in TB patients might help reducing it. This study investigates factors associated with mortality including patients' HIV sero-status, CD4 cell count, laboratory, nutritional and demographic characteristics in AFB smear positive pulmonary TB patients.

**Methods:**

We studied 887 sputum smear positive PTB patients, between 18 and 65 years of age receiving standard 8 months anti-TB treatment. Demographic, anthropometric and laboratory data including HIV, CD4 and other tests were collected at baseline and at regular intervals. Patients were followed for a median period of 2.5 years.

**Results:**

Of the 887 participants, 155 (17.5%) died, of whom 90.3% (140/155) were HIV-infected, a fatality of 29.7% (140/471) compared to 3.6% (15/416) among HIV-uninfected. HIV infection, age, low Karnofsky score, CD4 cell counts and hemoglobin, high viral load, and oral thrush were significantly associated with high mortality in all patients.

**Conclusion:**

Mortality among HIV-infected TB patients is high despite the use of effective anti-TB therapy. Most deaths occur after successful completion of therapy, an indication that patients die from causes other than TB. HIV infection is the strongest independent predictor of mortality in this cohort.

## Background

Worldwide, one out of three people are infected with Mycobacterium tuberculosis (MTB) [1]. Approximately 7 million new cases of TB and 1.7 million deaths due to TB were reported in 2006, the last year that global epidemiological data for TB is available [2]. The HIV epidemic has fuelled the current TB epidemic worldwide and in particular in sub-Saharan Africa [3].

HIV is the strongest factor in the development of active TB; it is estimated that only one out of ten immunocompetent persons infected with TB develops active TB in his/her lifetime; whereas, one out of ten HIV-infected persons infected with TB will develop active TB every year. Autopsy studies have shown that 30 to 40% of HIV-infected adults die from tuberculosis in Africa [4]. On the other hand TB has been shown to accelerate HIV disease progression to AIDS and probably early death [5-8].

If untreated, 50-80% of patients with smear-positive TB die; fortunately TB has an effective treatment. Current treatment regimens, given under appropriate management conditions, are nearly 100% curative for patients with drug-susceptible organisms. In a poorly implemented TB programme, as many as 30% of patients with smear-positive TB die; in contrast, death rates in direct observed treatment of TB (DOTS) programmes throughout the world are generally less than 5% [9].

Despite reported good and similar responses to anti-TB treatment in both HIV-infected and uninfected patients [10,11], mortality of HIV-infected TB patients during and following treatment is reported to be higher [12,13].

Appropriate management of factors that influences morbidity and mortality may assist in reducing mortality among TB patients receiving anti TB treatment. We conducted a study among patients with Acid Fast Bacilli (AFB) sputum smear-positive pulmonary TB in 5 TB treatment clinics in Dar es Salaam, Tanzania, to determine factors associated with mortality including patients' HIV sero-status, CD4 cell count, AFB concentration in sputum, nutritional, and demographic characteristics.

## Methods

### Study Population

The study population and recruitment methods have been described in detail earlier [14]. Briefly, 887 adults with pulmonary tuberculosis (TB) were enrolled in a randomized trial to examine the effects of micronutrient supplementation on TB treatment failure, relapse, and mortality. The trial started in April 2000 in Dar es Salaam, Tanzania and continued until April 2005. The eligibility criteria for the study included positive sputum smears for acid-fast bacilli (AFB), age between 18 and 65 years, Karnofsky performance score of ≥ 40% [15], plan to stay in Dar es Salaam for 2 years, not being pregnant, and not having received anti-TB treatment during the previous one year.

All patients received a daily combination of rifampicin, isoniazid, pyrazinamide, and ethambutol under direct observation of a health worker during the first 2 months (intensive phase) followed by 6 months of self-administered daily isoniazid and ethambutol, as per the Tanzania National TB and Leprosy Programme guidelines. Antiretroviral medications were not widely available to most HIV-infected persons in Tanzania at the time this trial was conducted.

A written informed consent was obtained from all the study participants. The institutional review boards of the Muhimbili University College of Health Sciences, the Tanzanian National AIDS Control Program, and the Harvard School of Public Health approved the study protocol.

### Assessment of Predictors and Outcome

At the time of initiation of anti-TB treatment, HIV status was assessed among consenting patients using 2 sequential ELISAs (Wellcozyme, Murex Biotech; Enzygnost anti-HIV1+2, Behring); discrepant results were resolved by Western Blot test (Genetic Systems). Both pre-test and post-test counselling was provided. Among HIV-infected patients, only those with hemoglobin concentration greater than 7 g/dL were enrolled in the trial.

At the time of randomization, research nurses collected information on various socio-demographic characteristics including age, education levels, marital status, and socioeconomic status. Anthropometric measurements were also obtained using standardized procedures [16] at the randomization visit as well as during each monthly follow-up visit. Height was measured to the nearest 0.1 cm using SECA Bodymeter 206 stadiometers, weight to the nearest 100 g in SECA 700 balance beam scales, and left mid-upper arm circumference (MUAC) at the midpoint between the acromion and olecranon to the nearest 0.1 cm using non-stretchable tailor's tapes.

A physician performed a complete physical examination and assessed HIV disease stage among the HIV-infected patients using the World Health Organization criteria [17]. The physician also recorded the presence of clinical signs and symptoms such as fever, nausea, diarrhea, fatigue, skin rashes, oral thrush, peripheral neuropathy, and extrapulmonary TB. A blood sample was also obtained for measurement of hemoglobin and albumin concentrations, CD4, CD3, and CD8 cell counts, using FACScount and FACSCAN systems (Becton Dickinson). Viral load was also determined using the Roche Amplicor v1.5 assay.

Treatment failure by 1 month was defined as positive AFB cultures 1 month after initiation of treatment. Early recurrence was defined as having a positive culture after 1 month after having tested culture negative by 1 month after treatment initiation. Late recurrence was defined as any positive culture after 8 months from treatment initiation. If any of the participants missed a follow-up visit, a research assistant visited his/her home to determine survival status. The median follow-up time for HIV-uninfected patients was 52 months (IQR: 47-57 months) and for HIV-infected patients was 30 months (IQR: 15-41 months).

### Data Management and Statistical Analysis

All information was double entered into Fox Pro databases, compared and corrected. Cox proportional hazards models were used to assess the relationship between various predictors and mortality. Mortality was assessed in three groups - overall in both HIV-infected and uninfected patients through the complete duration of follow-up; in HIV-infected patients alone; and in HIV-infected patients only in the first 8 months of follow-up. Variable with p-values less than 0.2 in univariate Cox regression analyses were included in the multivariate model; only the variables that had p-values less than 0.05 in this multivariate model were retained in the final model. All analyses were performed using SAS software version 9.2 (SAS Institute Inc., Cary).

## Results

The characteristics of the 887 patients included in this analysis are presented in Table 1. The mean age of patients who died was 36 years (± 8.5) compared to 31 years (± 8.8) for patients who were alive at the end of follow-up. 40% of the patients who died were female; 32% of the patients who were alive at the end of follow-up were female. More than 20% of those who died had Karnofsky scores less than 70% compared to only 9% of those who stayed alive. The mean hemoglobin at baseline was 9.6 (± 1.6) g/dL in those who died and 10.6 (± 1.8) g/dL in those who stayed alive. About 90% of those who died were HIV-infected; only 45% of those who stayed alive were HIV-infected. More than 62% of patients who died had CD4 cell counts lower than 200 cell/mm3, compared to only 11% of those who stayed alive. A greater proportion of the patients who died had chronic diarrhea, dysentery, hospital admissions, herpes zoster, oral ulcers, peripheral neuropathy, skin rash, and oral thrush compared to the patients who survived (Table 1).

**Table 1:** Characteristics of the Study Population

	Mortality	
	Yes (*n = 155*)	No (*n = 732*)
Characteristics	Mean (SD) or % (Number)	Mean (SD) or % (Number)
*Social and demographic variables*		
Age, yrs	35.97 (8.49)	31.35 (8.84)
Females	40.00% (62)	32.10% (235)
Karnofsky Score	71.74 (9.54)	75.17 (8.41)
Shillings* spent on food/person/day (quartiles)		
≤ 250	28.37% (40)	23.14% (149)
251-499	26.24% (37)	26.40% (170)
500-750	26.24% (37)	27.17% (175)
>750	19.15% (27)	23.29% (150)
***TB related***		
AFB Smear Result positive at baseline	96.75% (149)	94.12% (688)
AFB Culture positive at baseline	66.67% (44)	68.82% (287)
***Laboratory Results***		
Baseline Hemoglobin, g/dL	9.59 (1.59)	10.59 (1.82)
***HIV- or Immune function related***		
HIV infected	90.32% (140)	45.22% (331)
Baseline WHO HIV stage		
3	85.96% (98)	93.48% (215)
4	14.04% (16)	6.52% (15)
Baseline CD4 cell count/μL	238.15 (302.25)	564.04 (288.86)
Baseline CD3 cell count/μL	1086.23 (605.21)	1232.42 (501.09)
Baseline CD8 cell count/μL	785.61 (454.56)	601.85 (383.09)
CD4/CD8 Ratio	0.39 (0.71)	1.30 (0.90)
Log(10) viral load	5.11 (0.66)	4.38 (0.99)
Q1	6.45% (10)	11.61% (85)
Q2	14.19% (22)	9.97% (73)
Q3	18.06% (28)	9.15% (67)
Q4	34.84% (54)	5.60% (41)
***Clinical signs and symptoms***		
Diarrhea	9.45% (12)	10.12% (58)
Fatigue	71.65% (91)	66.32% (380)
Fever	55.12% (70)	64.22% (368)
Nausea/Vomiting	5.51% (7)	8.90% (51)
Skin Rash	20.47% (26)	8.90% (51)
Oral thrush	12.60% (16)	1.40% (8)
Weight loss	81.89% (104)	80.63% (462)
Extrapulmonary TB	1.57% (2)	2.44% (14)
***Anthropometry***		
BMI at enrolment, kg/m^2^	19.31 (3.04)	19.10 (2.64)
MUAC at enrolment, cm	23.25 (2.85)	23.25 (2.61)

* 1000 Tanzanian Shillings ≈ 1 US Dollar at the time of the trial

The univariate predictors (p < 0.20) of mortality in the cohort through the complete duration of follow-up are presented in Table 2. In the multivariate model (Table 3), each year increase in age was associated with a 4% higher risk of mortality (95% CI: 1.03, 1.06). Higher Karnofsky score and baseline hemoglobin concentrations were protective against mortality in this cohort. HIV infection was associated with a greater than 5 times increase in the risk of mortality over the follow-up period (95% CI; 2.55, 10.55). Patients with CD4 cell counts greater than 500 cells/mm3 had 80% lower risk of dying compared to patients with CD4 cell counts lower than 200 cells/mm3 (HR: 0.17; 95% CI: 0.09, 0.33). Similarly, patients in the highest quartile of viral load had 4 times the risk of dying compared to those in the lowest quartile (HR: 4.05; 95% CI: 2.01, 8.16). Patients with oral thrush at baseline also had a 2.5 times the risk of dying compared to the patients who did not (95% CI: 1.40, 4.28).

**Table 2:** Univariate Predictors of Mortality (p < 0.20)

	Univariate Analyses	
Characteristics	Hazard Ratio (HR)	p-value^1^
***Social and demographic variables***		
Age, years	1.05 (1.03, 1.06)	<0.01
Sex		
Females	1.45 (1.05, 2.00)	0.02
Karnofsky Score	0.96 (0.94, 0.97)	<0.01
Karnofsky Score <70%	2.43 (1.64, 3.60)	<0.01
Shillings spent on food/person/day	1 (0.999, 1)	0.09
***TB related***		
AFB Smear at baseline - Number of colonies		0.01
Negative	1	
1-9/100 fields	2.88 (0.94, 8.82)	
10-99/100 fields	2.15 (0.86, 5.37)	
1-10/field	2.22 (0.88, 5.62)	
>10/field	1.20 (0.47, 3.01)	
Early Relapse^2^	1.60 (0.80, 3.19)	0.18
Treatment failure 1 month post-treatment initiation	0.47 (0.25, 0.88)	0.02
Has ever had TB	0.70 (0.51, 0.97)	0.03
Received TB treatment in the past 5 years	1.96 (0.96, 4.00)	0.06
***Laboratory Results***		
Baseline Hemoglobin, g/dL	0.73 (0.67, 0.80)	<0.01
Baseline Albumin, g/L	0.83 (0.71, 0.97)	0.02
***HIV- or Immune function related***		
HIV infected	12.40 (7.24, 21.23)	<0.01
Baseline HIV stage		
3	1	
4	1.94 (1.14, 3.29)	0.01
Patient progressed from stage 3 to 4	1.91 (1.28, 2.84)	<0.01
Baseline CD4, per 100 cells/μL	0.57 (0.52, 0.63)	<0.01
CD4 cell count, cells/μL		<0.01
0-199	1	
200-499	0.14 (0.09, 0.22)	
>=500	0.04 (0.02, 0.07)	
Baseline CD3, per 100 cells/μL	0.94 (0.90, 0.98)	<0.01
Baseline CD8, per 100 cells/μL	1.10 (1.06, 1.14)	<0.01
CD4/CD8 Ratio	0.09 (0.05, 0.15)	<0.01
RNA Viral load, per 10,000 copies/ml	1.02 (1.02, 1.03)	<0.01
Log(10) viral load	2.70 (2.05, 3.54)	<0.01
Log(10) viral load, quartiles		<0.01
Q1	1	
Q2	2.54 (1.20, 5.37)	
Q3	3.67 (1.78, 7.56)	
Q4	8.63 (4.38, 17.00)	
***Clinical signs and symptoms***		
Depression >2 weeks, ever	1.45 (0.84, 2.52)	0.19
Chronic Diarrhea	7.88 (3.22, 19.29)	<0.01
Dysentery	2.22 (1.08, 4.53)	0.03
Fever	0.66 (0.47, 0.94)	0.02
Hospital admission	2.01 (0.94, 4.30)	0.07
Herpes Zoster, first episode	2.13 (0.79, 5.77)	0.14
Kaposi's Sarcoma	6.48 (1.60, 26.24)	0.01
Oral Ulcer	2.64 (1.16, 5.99)	0.02
Nausea/Vomiting	0.59 (0.28, 1.27)	0.18
Oropharyngeal candidiasis	5.16 (1.90, 13.99)	<0.01
Oral candidiasis	6.90 (4.07, 11.72)	<0.01
Peripheral Neuropathy	1.84 (1.04, 3.27)	0.04
Skin Rash	2.30 (1.49, 3.54)	<0.01
Oral thrush	7.35 (4.33, 12.48)	<0.01
***Anthropometry***		
Height, cm	0.97 (0.96, 0.99)	<0.01
Triceps Skinfold, average of 3 measurements	1.03 (1.00, 1.07)	0.04

^1 ^p-values obtained from Cox Regression Analyses

^2 ^Relapse between 1-8 months post-treatment initiation if culture negative at 1 month (Early Relapse)

**Table 3:** Multivariate Predictors of Mortality (p < 0.05)

	Multivariate Analyses	
Characteristics	Hazard Ratio (HR)	p-value^1^
Age, years	1.04 (1.03, 1.06)	<0.01
Karnofsky Score	0.98 (0.96, 1.00)	0.02
Baseline Hemoglobin, g/dL	0.87 (0.79, 0.96)	<0.01
HIV infected	5.18 (2.55, 10.55)	<0.01
CD4 groups, cells/μL		<0.01
0-199	1	
200-499	0.27 (0.17, 0.41)	
>=500	0.17 (0.09, 0.33)	
Log(10) viral load, quartiles		<0.01
Q1	1	
Q2	1.78 (0.84, 3.79)	
Q3	2.64 (1.27, 5.48)	
Q4	4.05 (2.01, 8.16)	
Oral thrush	2.45 (1.40, 4.28)	<0.01

^1 ^p-values obtained from Cox Regression Analyses

Among HIV-infected patients, greater age, lower Karnofsky score, lesser money spent on food every day, lower baseline hemoglobin, stage 4 HIV disease, lower CD4 and CD3 cell counts, higher viral load, and having chronic diarrhea, oral ulcer, peripheral neuropathy, skin rash, and oral thrush were all associated with increased risk of mortality in univariate analyses (data not shown). In the multivariate model (Table 4), greater age, lower Karnofsky score, baseline CD3 cell count, and CD4/CD8 ratio, higher viral load at baseline, and having oral thrush at baseline were independent significant predictors of increased mortality. Recruitment at Temeke center was associated with a significantly higher risk of mortality compared to the other four centers. The presence of nausea/vomiting at baseline was associated with reduced mortality in this HIV-infected cohort.

**Table 4:** Multivariate Predictors of Mortality among HIV-infected patients (p < 0.05)

	Multivariate Analyses	
Characteristics	Hazard Ratio (HR)	p-value^1^
Age, years	1.03 (1.01, 1.05)	0.01
Center		0.01
Mwananyamala	0.50 (0.31, 0.82)	
Temeke	1	
Tandale	0.57 (0.36, 0.92)	
Mbgala	0.94 (0.51, 1.74)	
Amana	0.50 (0.28, 0.89)	
Karnofsky Score	0.98 (0.96, 0.99)	0.01
Baseline CD3, per 100 cells/μL	0.95 (0.92, 0.99)	0.01
CD4/CD8 Ratio, per 0.1 change	0.61 (0.54, 0.70)	<0.01
Log(10) viral load, quartiles		<0.01
Q1	1	
Q2	1.52 (0.71, 3.27)	
Q3	2.60 (1.23, 5.49)	
Q4	3.17 (1.57, 6.41)	
Nausea/Vomiting	0.28 (0.10, 0.84)	0.02
Oral thrush	3.17 (1.76, 5.72)	<0.01

^1 ^p-values obtained from Cox Regression Analyses

When analyses was restricted to the first 8 months of follow-up (the treatment phase) in the HIV-infected patients in this cohort, the univariate predictors of mortality remained largely similar (data not shown). In the multivariate model (Table 5), however, viral load and nausea/vomiting did not remain as predictors. Height group (in quartiles) was found to be a significant independent predictor of mortality; the patients in quartile 3 had a 2.6 times higher risk of mortality compared to those in quartile 1 (95% CI: 1.01, 6.70).

**Table 5:** Multivariate Predictors of Mortality by 8 months among HIV-infected patients (p < 0.05)

	Multivariate Analyses	
Characteristics	Hazard Ratio (HR)	p-value^1^
Age, years	1.05 (1.01, 1.09)	0.01
Karnofsky Score, <70%	2.40 (1.16, 4.98)	0.02
Baseline CD3, per 100 cells/μL	0.92 (0.85, 0.98)	0.02
CD4/CD8 Ratio, per 0.1 change	0.51 (0.35, 0.72)	<0.01
Oral thrush	3.87 (1.54, 9.72)	<0.01
Height group, quartiles		0.03
<158.1 cm	1	
158.1-164.0 cm	1.95 (0.83, 4.57)	
164.1-169.5 cm	2.60 (1.01, 6.70)	
≥ 169.6 cm	0.41 (0.11, 1.51)	

^1 ^p-values obtained from Cox Regression Analyses

## Discussion

Mortality among TB patients infected with HIV during and following completion of anti-TB treatment in the absence of ART remains high as confirmed by this study. Older age, HIV infection, lower Karnofsky scores, hemoglobin levels, and CD4 cell counts, higher viral load, and oral thrush were independent predictors of mortality in this analysis. Among HIV-infected patients, older age, lower Karnofsky score, CD3 cell counts, and CD4/CD8 ratio, higher viral load, recruitment at Temeke, and oral thrush were independent predictors of mortality. Presence of nausea/vomiting was protective against death in these patients.

The overall mortality of 17.5% in this study was mainly contributed by a high mortality rate among HIV-infected patients (29.7%) as opposed to comparatively low mortality (3.6%) among HIV-uninfected patients; over 90% (140/155) of the deaths were among the HIV-infected patients. This high mortality occurred despite the use of a highly effective Rifampicin containing DOTS regimen for 2 months as part of initial intensive therapy. Similar high mortality rates during and after TB treatment among HIV-infected patients with smear-positive pulmonary TB have been reported in a number of studies in sub Saharan Africa [18]. Harries et al estimated that in sub Saharan Africa, up to 30% of HIV-infected TB patients die within 12 months of starting anti TB treatment [7]. The mortality rate among HIV-infected patients with TB, particularly among those with no access to anti-retroviral treatment (ART), is similar in other parts of the world as well. For example, in a study in Cambodia, 27% of HIV-infected patients with TB died during follow-up [19]. In a large trial in US and Canada, 29.6% of HIV-infected TB patients who did not receive any ART died during follow-up (28 months) [20].

Excess deaths in HIV-infected TB patients in this study were probably caused by conditions other than TB, most likely by HIV-related causes [7]. While deaths during treatment may be considered to be due to TB, deaths occurring after successful completion of TB treatment can be considered to be due to factors other than TB such as HIV-related causes. In this study only 25.7% (36/140) of deaths in the HIV-infected patients occurred during the 8 months of anti-TB therapy while the rest 74.3% (104/140) occurred after successful completion of their therapy. Although the cause of mortality is difficult to confirm in the absence of autopsy, as was the case in this study, the presence of advanced HIV disease in patients receiving potent anti-TB therapy, suggests that deaths in most cases occurred from diseases other than TB. Therefore, it is extremely important to ensure delivery of HAART to HIV-infected patients with TB. Several studies have shown a dramatic decline in mortality rates with introduction of HAART in this group of patients. For example, age- and sex-standardized mortality rates decreased from 22.9% to 11.8% between 1993-95 and 1999-2001 in the Netherlands, probably because of widespread availability of HAART by 1999 [21]. Other studies have also shown that HAART may reduce the mortality in HIV-infected TB patients by as much as 60% [22]. Only 10.5% of the patients who received HAART died during follow-up compared to 29.6% of those who did not receive any ART in a study from North America [20].

Older age is a well-established risk factor for mortality among TB patients [23-27]. For example, patients 55 years or older had a 2.4 times increased risk of dying in multivariate analyses, compared to patients between the ages of 15-24 in a study in Guinea-Bissau [12]. Similarly, in a study in Singapore, older age (≥ 65 years) was associated with 8 times the risk of mortality compared to younger patients (<45 years) [28].

HIV sero-status has been reported to be the most important factor associated with mortality both during and after treatment among patients with smear positive pulmonary TB [20,23,25,29-31]. In a study from Guinea-Bissau, HIV-1 infection was associated with approximately 5 times greater risk of mortality compared to uninfected TB patients [12]; this effect size is similar to our results. Low CD4 cell counts have also been shown to increase the risk of mortality among TB patients [32]; in a study in Uganda, low CD4 cell count at baseline among HIV-infected TB patients initiating ART was the only significant independent predictor of mortality [33].

The presence of TB in HIV infected patients is likely to accelerate the progression of HIV disease. TB infection and disease stimulates the host immune system including replication of T-lymphocytes, which subsequently enhances viral replication leading to high viral load. Low CD4 cell counts coupled with a high viral load are associated with faster HIV disease progression making patients susceptible to opportunistic illnesses, which may have been the cause of death despite successful TB treatment. Similarly, Karnofsky score could represent how sick the patients were at baseline; lower Karnofsky score implies advanced disease and thus may explain its association with higher risk of death. Presence of diseases such as oral thrush [12,32] also indicates immunocompromise in the patient and suggests that these patients had more advanced disease at baseline, leading to greater mortality.

Recruitment at the Temeke center could possibly be explained by it being a bigger hospital and hence attracting sicker patients from adjoining areas. The presence of nausea/vomiting being protective against mortality in HIV-infected TB patients is novel and we did not find any studies presenting a similar association.

We also found that lower baseline hemoglobin was associated with mortality in this group of patients. This is in accordance with other studies which have found a similar association; for example, a study in Samara, Russia, found that anemia (defined as hemoglobin <12 g/dL in males and <11 g/dL in females) was associated with 5 times greater odds of dying among TB patients [34]. In another study conducted in Malawi and Zambia, the investigators found that hemoglobin levels >10 g/dl in HIV-infected patients with TB were associated with almost 80% reduction in risk of mortality during and after anti-TB treatment [35]. In our earlier work, we have shown that anemia is an independent risk factor of mortality in HIV-infected patients in Tanzania [36]; this has also been shown in studies from Europe [37].

Measures to prevent deaths in TB patients in countries with high HIV prevalence should include the use of; 1) antiretroviral therapy (ART), which is likely to have the greatest impact; 2) TB treatment regimens of proven effectiveness; 3) preventive therapy for HIV-related diseases other than TB; 4) improved TB and HIV control services; and 5) improved general health services with better diagnosis and treatment of HIV-related diseases [8].

The wide spread use of ART is expected to be associated with the reduction in mortality from HIV related diseases including TB. Irrespective of the proportions of deaths that are due to TB or to other HIV-related diseases, TB deaths in high HIV prevalence populations represent a joint failure of TB and HIV programs. National TB Programs face the challenge not only of ensuring the effective diagnosis and treatment of increasing numbers of TB patients, but also of trying to identify and implement ways of reducing TB deaths in collaboration with HIV programmes [6,7].

## Conclusion

This study has demonstrated that

1. Mortality among TB patients co-infected with HIV is still high in the absence of ART despite adequate treatment using potent anti-TB drugs.

2. Most patients died after successful completion of their anti TB therapy indicating that TB was less likely to be the cause of death.

3. HIV sero-status, older age, Karnofsky score, low hemoglobin levels and CD4 cell counts, higher viral load, and oral thrush at baseline are predictors of mortality among TB patients.

4. Older age, Karnofsky score, low CD3 cell counts and CD4/CD8 ratio, high viral load, oral thrush, and absence of nausea/vomiting at baseline are predictors of mortality among TB and HIV co-infected patients.

## Competing interests

The authors declare that they have no competing interests.

## Authors' contributions

FMM and SM wrote the initial draft of the manuscript; SM and RJB contributed to the data analysis; FMM, EV, WU, ES, and WWF were investigators on the trial and contributed to design and implementation as well as data collection; all authors contributed to interpretation of data and manuscript preparation.

All authors have read and approved the final manuscript.

## Pre-publication history

The pre-publication history for this paper can be accessed here:

http://www.biomedcentral.com/1471-2458/9/409/prepub

## References

[B1] Narain JP Lo YR Epidemiology of HIV-TB in Asia Indian J Med Res 2004 120 4 277 289 15520482

[B2] WHO Global Tuberculosis Control: surveillance, planning, financing 2008 Geneva: World Health Organization

[B3] Chaisson RE Martinson NA Tuberculosis in Africa--combating an HIV-driven crisis N Engl J Med 2008 358 11 1089 1092 10.1056/NEJMp0800809 18337598

[B4] Ansari NA Kombe AH Kenyon TA Hone NM Tappero JW Nyirenda ST Binkin NJ Lucas SB Pathology and causes of death in a group of 128 predominantly HIV-positive patients in Botswana, 1997-1998 Int J Tuberc Lung Dis 2002 6 1 55 63 11931402

[B5] Maniar JK Kamath RR Mandalia S Shah K Maniar A HIV and tuberculosis: partners in crime Indian J Dermatol Venereol Leprol 2006 72 4 276 282 10.4103/0378-6323.26723 16880573

[B6] Maher D Watt CJ Williams BG Raviglione M Dye C Tuberculosis deaths in countries with high HIV prevalence: what is their use as an indicator in tuberculosis programme monitoring and epidemiological surveillance? Int J Tuberc Lung Dis 2005 9 2 123 127 15732729

[B7] Harries AD Hargreaves NJ Kemp J Jindani A Enarson DA Maher D Salaniponi FM Deaths from tuberculosis in sub-Saharan African countries with a high prevalence of HIV-1 Lancet 2001 357 9267 1519 1523 10.1016/S0140-6736(00)04639-0 11377627

[B8] Mukadi YD Maher D Harries A Tuberculosis case fatality rates in high HIV prevalence populations in sub-Saharan Africa AIDS 2001 15 2 143 152 10.1097/00002030-200101260-00002 11216921

[B9] Frieden TR Can tuberculosis be controlled? Int J Epidemiol 2002 31 5 894 899 10.1093/ije/31.5.894 12435756

[B10] Okwera A Whalen C Byekwaso F Vjecha M Johnson J Huebner R Mugerwa R Ellner J Randomised trial of thiacetazone and rifampicin-containing regimens for pulmonary tuberculosis in HIV-infected Ugandans. The Makerere University-Case Western University Research Collaboration Lancet 1994 344 8933 1323 1328 10.1016/S0140-6736(94)90693-9 7526098

[B11] Elliott AM Halwiindi B Hayes RJ Luo N Mwinga AG Tembo G Machiels L Steenbergen G Pobee JO Nunn PP The impact of human immunodeficiency virus on response to treatment and recurrence rate in patients treated for tuberculosis: two-year follow-up of a cohort in Lusaka, Zambia J Trop Med Hyg 1995 98 1 9 21 7861484

[B12] Gustafson P Gomes VF Vieira CS Samb B Naucler A Aaby P Lisse I Clinical predictors for death in HIV-positive and HIV-negative tuberculosis patients in Guinea-Bissau Infection 2007 35 2 69 80 10.1007/s15010-007-6090-3 17401710

[B13] Corbett EL Watt CJ Walker N Maher D Williams BG Raviglione MC Dye C The growing burden of tuberculosis: global trends and interactions with the HIV epidemic Arch Intern Med 2003 163 9 1009 1021 10.1001/archinte.163.9.1009 12742798

[B14] Villamor E Mugusi F Urassa W Bosch RJ Saathoff E Matsumoto K Meydani SN Fawzi WW A Trial of the Effect of Micronutrient Supplementation on Treatment Outcome, T Cell Counts, Morbidity, and Mortality in Adults with Pulmonary Tuberculosis J Infect Dis 2008 197 11 1499 1505 18471061 10.1086/587846 PMC2564793

[B15] Karnofsky DA Abelmann WH Craver LF Burchenal JH The use of nitrogen mustards in the palliative treatment of cancer Cancer 1948 1 634 656 10.1002/1097-0142(194811)1:4<634::AID-CNCR2820010410>3.0.CO;2-L

[B16] Lohman TG Roche AF Martorell R Anthropometric standardization reference manual 1988 Champaign, IL: Human Kinetics Books

[B17] WHO Interim proposal for a WHO staging system for HIV infection and disease Wkly Epidemiol Rec 1990 65 221 224 1974812

[B18] Mukadi YDMD Harries A Tuberculosis case fatality rates in high HIV prevalence populations in sub-Saharan Africa AIDS 2001 15 143 152 10.1097/00002030-200101260-00002 11216921

[B19] Cain KP Kanara N Laserson KF Vannarith C Sameourn K Samnang K Qualls ML Wells CD Varma JK The epidemiology of HIV-associated tuberculosis in rural Cambodia Int J Tuberc Lung Dis 2007 11 9 1008 1013 17705980

[B20] Sterling TR Zhao Z Khan A Chaisson RE Schluger N Mangura B Weiner M Vernon A Consortium TT Mortality in a large tuberculosis treatment trial: modifiable and non-modifiable risk factors Int J Tuberc Lung Dis 2006 10 5 542 549 16704037

[B21] Haar CH Cobelens FG Kalisvaart NA van Gerven PJ Have JJ van der HIV-related mortality among tuberculosis patients in The Netherlands, 1993-2001 Int J Tuberc Lung Dis 2007 11 9 1038 1041 17918662

[B22] Dheda K Lampe FC Johnson MA Lipman MC Outcome of HIV-associated tuberculosis in the era of highly active antiretroviral therapy J Infect Dis 2004 190 9 1670 1676 10.1086/424676 15478074

[B23] Zachariah R Spielmann MP Harries AD Salaniponi FM Moderate to severe malnutrition in patients with tuberculosis is a risk factor associated with early death Trans R Soc Trop Med Hyg 2002 96 3 291 294 10.1016/S0035-9203(02)90103-3 12174782

[B24] Lubart E Lidgi M Leibovitz A Rabinovitz C Segal R Mortality of patients hospitalized for active tuberculosis in Israel Isr Med Assoc J 2007 9 12 870 873 18210928

[B25] de Albuquerque MdeF Ximenes RA Lucena-Silva N de Souza WV Dantas AT Dantas OM Rodrigues LC Factors associated with treatment failure, dropout, and death in a cohort of tuberculosis patients in Recife, Pernambuco State, Brazil Cadernos de saúde pública/Ministério da Saúde, Fundação Oswaldo Cruz, Escola Nacional de Saúde Pública 2007 23 7 1573 1582 10.1590/s0102-311x2007000700008 17572806

[B26] Domingos MP Caiaffa WT Colosimo EA Mortality, TB/HIV co-infection, and treatment dropout: predictors of tuberculosis prognosis in Recife, Pernambuco State, Brazil Cadernos de saúde pública/Ministério da Saúde, Fundação Oswaldo Cruz, Escola Nacional de Saúde Pública 2008 24 4 887 896 10.1590/s0102-311x2008000400020 18392367

[B27] Zevallos M Justman JE Tuberculosis in the elderly Clin Geriatr Med 2003 19 1 121 138 10.1016/S0749-0690(02)00057-5 12735118

[B28] Low S Ang LW Cutter J James L Chee CB Wang YT Chew SK Mortality among tuberculosis patients on treatment in Singapore Int J Tuberc Lung Dis 2009 13 3 328 334 19275792

[B29] Glynn JR Warndorff DK Fine PE Munthali MM Sichone W Ponnighaus JM Measurement and determinants of tuberculosis outcome in Karonga District, Malawi Bull World Health Organ 1998 76 3 295 305 9744250 PMC2305706

[B30] Elliott AM Halwiindi B Hayes RJ Luo N Mwinga AG Tembo G Machiels L Steenbergen G Pobee JO Nunn P The impact of human immunodeficiency virus on mortality of patients treated for tuberculosis in a cohort study in Zambia Trans R Soc Trop Med Hyg 1995 89 1 78 82 10.1016/0035-9203(95)90668-1 7747316

[B31] Quy HT Cobelens FG Lan NT Buu TN Lambregts CS Borgdorff MW Treatment outcomes by drug resistance and HIV status among tuberculosis patients in Ho Chi Minh City, Vietnam Int J Tuberc Lung Dis 2006 10 1 45 51 16466036

[B32] Reyes-Corcho A Capo de Paz V Díaz-Jidy M Pérez-Avila J Bouza-Jiménez Y [Change in the survival of Cuban AIDS patients with tuberculosis in the Highly Active Antiretroviral Therapy (HAART) era] Investigación clínica 2008 49 3 309 320 18846772

[B33] Moore D Liechty C Ekwaru P Were W Mwima G Solberg P Rutherford G Mermin J Prevalence, incidence and mortality associated with tuberculosis in HIV-infected patients initiating antiretroviral therapy in rural Uganda AIDS 2007 21 6 713 719 10.1097/QAD.0b013e328013f632 17413692

[B34] Kourbatova EV Borodulin BE Borodulina EA del Rio C Blumberg HM Leonard MK Risk factors for mortality among adult patients with newly diagnosed tuberculosis in Samara, Russia Int J Tuberc Lung Dis 2006 10 11 1224 1230 17131780

[B35] Ciglenecki I Glynn JR Mwinga A Ngwira B Zumla A Fine PE Nunn A Population differences in death rates in HIV-positive patients with tuberculosis Int J Tuberc Lung Dis 2007 11 10 1121 1128 17945070

[B36] O'Brien ME Kupka R Msamanga GI Saathoff E Hunter DJ Fawzi WW Anemia is an independent predictor of mortality and immunologic progression of disease among women with HIV in Tanzania J Acquir Immune Defic Syndr 2005 40 2 219 225 10.1097/01.qai.0000166374.16222.a2 16186741

[B37] Mocroft A Kirk O Barton SE Dietrich M Proenca R Colebunders R Pradier C dArminio Monforte A Ledergerber B Lundgren JD Anaemia is an independent predictive marker for clinical prognosis in HIV-infected patients from across Europe. EuroSIDA study group Aids 1999 13 8 943 950 10.1097/00002030-199905280-00010 10371175

